# Impact of trazodone once‐a‐day on quality of life and functional recovery in adults with major depressive disorder: A prospective, observational study

**DOI:** 10.1002/brb3.3580

**Published:** 2024-07-21

**Authors:** Valeria Tellone, Oto Markovic, Milena Strashimirova, Gabriele Sani, William R. Lenderking, Mary Kay Margolis, Raffaella Fallone, Elisa Quarchioni, Agnese Cattaneo, Alessandro Comandini

**Affiliations:** ^1^ Global Medical Department Angelini Pharma S.p.A. Rome Italy; ^2^ Clinline Services s.r.o. Stredoceský kraj Czech Republic; ^3^ Diagnostic Consultative Center 14 Hospital VITA Sofia Bulgaria; ^4^ Department of Neuroscience, Section of Psychiatry Università Cattolica del Sacro Cuore Rome Italy; ^5^ Department of Neuroscience, Sensory Organs and Thorax, UOC Psichiatria Clinica e D'Urgenza Fondazione Policlinico Universitario A Gemelli IRCCS Rome Italy; ^6^ Evidera‐PPD Bethesda Maryland USA; ^7^ Pharmacometrics & Clinical Supply Angelini Pharma S.p.A. Rome Italy

**Keywords:** health‐related quality of life (HRQL), major depressive disorder, selective serotonin reuptake inhibitors, trazodone

## Abstract

**Background:**

Health‐related quality of life (HRQL) is an important goal for patients with major depressive disorder (MDD), but whether antidepressants improve HRQL in these patients is unclear. Here, we describe the real‐world effects of trazodone once‐a‐day (TzOAD) and selective serotonin reuptake inhibitor (SSRI) treatments on HRQL and functioning in adults with MDD.

**Methods:**

This 8‐week prospective, observational, open‐label, multicenter study was conducted in adults with moderate or severe MDD for whom TzOAD or SSRI were prescribed as monotherapy. The primary outcome was life enjoyment and satisfaction assessed via the patient‐reported Quality‐of‐Life Enjoyment and Satisfaction Questionnaire Short Form (Q‐LES‐Q‐SF) from baseline to week 8. Secondary outcomes included change in Q‐LES‐Q‐SF from baseline to weeks 1 and 2; severity of depressive symptoms using the Montgomery Åsberg Depression Rating Scale (MADRS) and sleep disturbance via the PROMIS SF‐SD 8b questionnaire at weeks 1, 2, and 8; and overall functioning via the Sheehan Disability Scale (SDS), hedonic capacity using the Snaith–Hamilton Pleasure Scale (SHAPS), and cognitive dysfunction using the Perceived Deficits Questionnaire (PDQ‐5) at baseline and week 8.

**Results:**

The study included 208 adults with MDD (mean [SD] age = 50.2 [14.3] years; 68.6% female; 98.4% White). Life enjoyment and satisfaction improved from baseline to week 8 for both treatment groups: Q‐LES‐Q‐SF mean (SD) scores were 27.5 (20.4) for the SSRI group and 39.0 (22.1) for the TzOAD group. Depressive symptoms and sleep disturbances also reduced from baseline to week 8: MADRS (SSRI, −15.7 [8.3]; TzOAD, −21.0 [9.8]); PROMIS SF‐SD 8b (SSRI, −9.9 [12.6]; TzOAD, −22.0 [12.6]). Mean change scores in Q‐LES‐Q‐SF, MADRS, and PROMIS SF‐SD 8b improved as early as week 1 in both groups. Mean scores also improved from baseline to week 8 on SDS (SSRI, −9.2 [7.4]; TzOAD, −14.3 [7.5]), SHAPS (SSRI, −6.6 [4.3]; TzOAD, −8.3 [4.4]), and PDQ‐5 (SSRI, −5.8 [4.5]; TzOAD, −7.7 [5.0]).

**Conclusions:**

In adults with MDD who received TzOAD or SSRIs, overall and individual HQRL domains improved rapidly and in parallel with improvements in depressive symptoms, with a slightly greater improvement observed in the TzOAD group.

## Graphical abstract

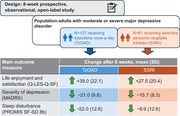

## 1 INTRODUCTION

Major depressive disorder (MDD) is a severe mood disorder that is estimated to affect 4.7% of the global population annually (Ferrari et al., 2013). MDD is characterized by depressed mood or anhedonia (i.e., loss of pleasure), with at least three additional symptoms, for at least ≥2 consecutive weeks and is associated with an increased risk of chronic diseases (American Psychiatric Association, 2013; Chesney et al., 2014; Cuijpers et al., 2013; Diniz et al., 2013; Hsu et al., 2016; Katon et al., 2010). The World Health Organization has projected that MDD will rank first in contributing to the global disease burden by 2030 (WHO, 2004). Patients with MDD experience an impaired health‐related quality of life (HRQL), including persistently depressed mood, loss of pleasure, disturbed sleep, feelings of guilt and low self‐worth, and cognitive deficits (Daly et al., 2010; Demyttenaere et al., 2002; Judd et al., 2000; Papakostas et al., 2004; Proudman et al., 2021; Suthoff et al., 2022). MDD affects an individual's ability to perform daily activities including work. Kessler et al. (2003) found that >96% of patients who had MDD for >1 year reported not being able to work or perform daily activities for an average of 35.2 days within the past year (Cambridge et al., 2018; Knight & Baune, 2018; Kessler et al., 2003; McIntyre & Lee, 2016). In 2019, MDD accounted for 1.47% of all disability‐adjusted life‐years worldwide (GBD 2019 Diseases and Injuries Collaborators 2020).

Patients with MDD experience persistently depressed mood, anhedonia, disturbed sleep, feelings of guilt and low self‐worth, and cognitive deficits (Judd et al., 2000; Demyttenaere et al., 2002; Papakostas et al., 2004; Daly et al., 2010; Proudman et al., 2021; Suthoff et al., 2022). Mood symptoms of MDD are associated with reduced physical, psychological, and social functioning, and contribute to a reduced health‐related quality of life (HRQL) (Kessler et al., 2003; Papakostas et al., 2004; Trivedi et al., 2006; Israel 2010). Patients with MDD in remission after antidepressant therapy may continue to experience poorer mental and physical functioning than the general population (Israel 2010).

Selective serotonin reuptake inhibitors (SSRIs) and serotonin norepinephrine reuptake inhibitors (SNRIs), along with cognitive behavioral therapy, are considered as first‐line treatments for MDD (Agius & Bonnici, 2017). Delayed onset of action is a limitation of some antidepressant treatments (e.g., SSRIs and SNRIs), which may lead to noncompliance, poor outcomes, and treatment failures (Nierenberg et al., 1999; Trivedi et al., 2006). Furthermore, nearly a third of patients with MDD discontinue treatment due to adverse effects (Demyttenaere & Haddad, 2000; Gollan et al., 2012; Hu et al., 2004; Solmi et al., 2021). Although SSRIs and SNRIs are known to improve depressive symptoms and HRQL in patients with MDD (Hofmann et al., 2017), relatively few clinical studies have used validated scales for patient‐reported outcomes to evaluate the magnitude and timing of these improvements.

Trazodone hydrochloride is a triazolopyridine derivative synthetized in the 1960s and is the first serotonin receptor antagonist and reuptake inhibitor developed and approved for the treatment of depression with or without anxiety in adults (Fagiolini et al., 2012; Sheehan et al., 2009). Trazodone simultaneously inhibits serotonin transporter and serotonin type 2 (5‐HT_2A_ and 5‐HT_2C_) receptors and moderately inhibits histamine‐1 receptor activity (Sheehan et al., 2009). The trazodone once‐a‐day (TzOAD) formulation releases the active ingredient over 24 h, with the goal of improving treatment adherence and tolerability (Sheehan et al., 2009). TzOAD is well tolerated and is less likely than SSRIs and SNRIs to cause weight gain and sexual dysfunction (Clayton et al., 2006; Fava, 2004; Hidalgo & Sheehan, 2010; Thompson, 2002; Uguz et al., 2015). Trazodone is reported to improve sleep (Sheehan et al., 2009), but its effect on HRQL in patients with MDD has not been well documented. The current study used validated scales to examine the real‐world effect of TzOAD and SSRI monotherapy on overall and individual domains of HRQL in adults with MDD.

## 2 METHODS

### 2.1 Study design

This was an 8‐week, prospective, observational, open‐label study conducted at 19 sites in Bulgaria, Czech Republic, and Italy between December 2021 and August 2022. The study enrolled adults (≥18 years) with moderate or severe MDD (Montgomery Åsberg Depression Rating Scale [MARDS] scores at baseline ≥20) (Montgomery & Asberg, 1979) for whom TzOAD or any available SSRI was prescribed as antidepressant monotherapy based on their physician's discretion. TzOAD and SSRI dosage and treatment had to have been used in accordance with the local summary of product characteristics and clinical practice. Patients treated with a combination of antidepressant drugs or with other drugs aiming to improve mood (e.g., antipsychotics and mood stabilizers) were excluded from the study. To participate, patients had to provide written informed consent.

**TABLE 1:** Clinical outcome assessments.

Assessment	Description	Score
Q‐LES‐Q‐SF	The Q‐LES‐Q‐SF is 16‐item questionnaire that evaluates the patient's overall joy and satisfaction with physical health, mood, occupation, housework, social relationships, family relationships, leisure activities, daily functioning, sexual life, economic status, living conditions, the possibility of physical activity without feeling dizzy and/or unstable, sight during work or with hobbies, general well‐being, and medication.	Patients select a response from “very poor” (score = 1) to “very good” (score = 5). Responses are summed to obtain a total score (range 14–70). Total scores are then converted to a scale of 0–100 so that the scores represent a percentage of the maximum possible score. Higher scores indicate better enjoyment and satisfaction with life.
PROMIS SF‐SD 8b	The PROMIS SF‐SD 8b is an 8‐item patient‐reported measure evaluating sleep disturbance in the 7 days preceding the assessment. Sleep quality, satisfaction, and difficulty with falling and staying asleep are evaluated on a five‐point Likert scale.	Raw scores are converted to a standardized *t*‐score using the PROMIS Scoring Manual. Higher scores indicate greater sleep disturbance.
SDS	The SDS assesses three domains of functioning (work/school, social life/leisure, and family life) in the 7 days preceding the assessment.	Domain scores range from 0 to 10, with higher scores indicating greater functional impairment.
SHAPS	The SHAPS is a 14‐item patient‐reported measure of hedonic capacity.	Each item is scored on a response scale ranging from “definitely agree” to “strongly disagree.” The total score is a sum of each of the 14 items and ranges from 0 to 14. Higher total scores indicate a higher level of anhedonia.
PDQ‐5	The PDQ‐5 is a 5‐item questionnaire that assesses cognitive dysfunction in people with depression. Items measured include attention/concentration, retrospective memory, prospective memory, and planning/organization over the four weeks preceding the assessment.	The total score ranges from 0 to 20. Higher scores indicate greater perceived cognitive dysfunction.
MADRS	The MADRS is a 10‐item clinician‐rated scale. The 10 items were selected for their ability to detect changes due to antidepressant treatment and their high correlations with overall change in depression. The MADRS is used in patients with MDD to measure the overall severity of depressive symptoms.	Items are rated on a scale of 0–6. The total score is the sum of the items (range 0–60). Higher scores indicate greater severity of depressive symptoms.

Abbreviations: MADRS, Montgomery Åsberg Depression Rating Scale; MDD, major depressive disorder; PDQ‐5, Perceived Deficits Questionnaire; PROMIS SF‐SD 8b, Patient‐Reported Outcomes Measurement Information System Sleep Disturbance Short Form 8b; Q‐LES‐Q‐SF, Quality of Life Enjoyment and Satisfaction Questionnaire Short Form; SDS, Sheehan Disability Scale; SHAPS, Snaith–Hamilton Pleasure Scale.

The study was approved by the relevant regulatory authorities and ethics committees. The study was carried out in accordance with the International Conference on Harmonisation guidelines on Good Clinical Practice and local ethical and legal requirements and regulations. Confidential patient information was treated in accordance with the Declaration of Helsinki, Regulation (EU) 2016/679, and applicable local data protection laws.

### 2.2 Statistical analysis

A projected full analysis set of 165 patients was anticipated based on an estimate that 206 patients with MDD would initiate antidepressant monotherapy with TzOAD or any available SSRI at the study sites, combined with a 20% drop‐out rate. All calculations were performed on the full analysis set, which included all patients who had baseline and at least one post‐baseline endpoint assessment.

Analyses were performed using SAS Enterprise Guide version 7.1 (SAS Institute). This study was descriptive, and no formal hypothesis testing was performed.

The primary endpoint was the change in life enjoyment and satisfaction, as assessed by the Quality‐of‐Life Enjoyment and Satisfaction Questionnaire Short Form (Q‐LES‐Q‐SF), from baseline to week 8. The Q‐LES‐Q‐SF is a patient‐reported outcome commonly used to measure HRQL in patients with depression and anxiety disorders in clinical research (Stevanovic 2011). Patient demographics, co‐morbidities, current psychiatric status, and treatment, medical, MDD, and psychiatric histories were collected at baseline. Secondary endpoints included change in the Q‐LES‐Q‐SF score from baseline to weeks 1 and 2; change in the Patient‐Reported Outcomes Measurement Information System Sleep Disturbance Short Form 8b (PROMIS SF‐SD 8b) (Health Measures 2013) and MADRS scores from baseline to weeks 1, 2, and 8; change in patient‐reported overall functioning (i.e., work/school, social life/leisure, and family life) evaluated using the Sheehan Disability Scale (SDS) scores (Sheehan et al., 1996) from baseline to week 8; change in hedonic capacity assessed using the Snaith‐Hamilton Pleasure Scale (SHAPS) (Snaith et al., 1995) from baseline to week 8; and change in cognitive dysfunction evaluated using the Perceived Deficits Questionnaire (PDQ‐5) (Sullivan et al., 1990) from baseline to week 8; percent of enrolled patients who discontinued the study before completing all visits; and percent of enrolled patients on treatment at week 8. All outcome measures are summarized in Table 1.

Spearman correlation coefficients were calculated comparing Q‐LES‐Q‐SF scores with MADRS and PROMIS SF‐SD 8b scores, as well as MADRS scores with PROMIS SF‐SD 8b scores at baseline and weeks 1, 2, and 8. Change score correlations were calculated at each post‐baseline timepoint for each measure. Correlations <.30 were considered weak, between >.30 and ≤.50 were considered moderate, and >.50 were considered strong (Cohen, 1988).

## 3 RESULTS

### 3.1 Study population

**FIGURE 1:**
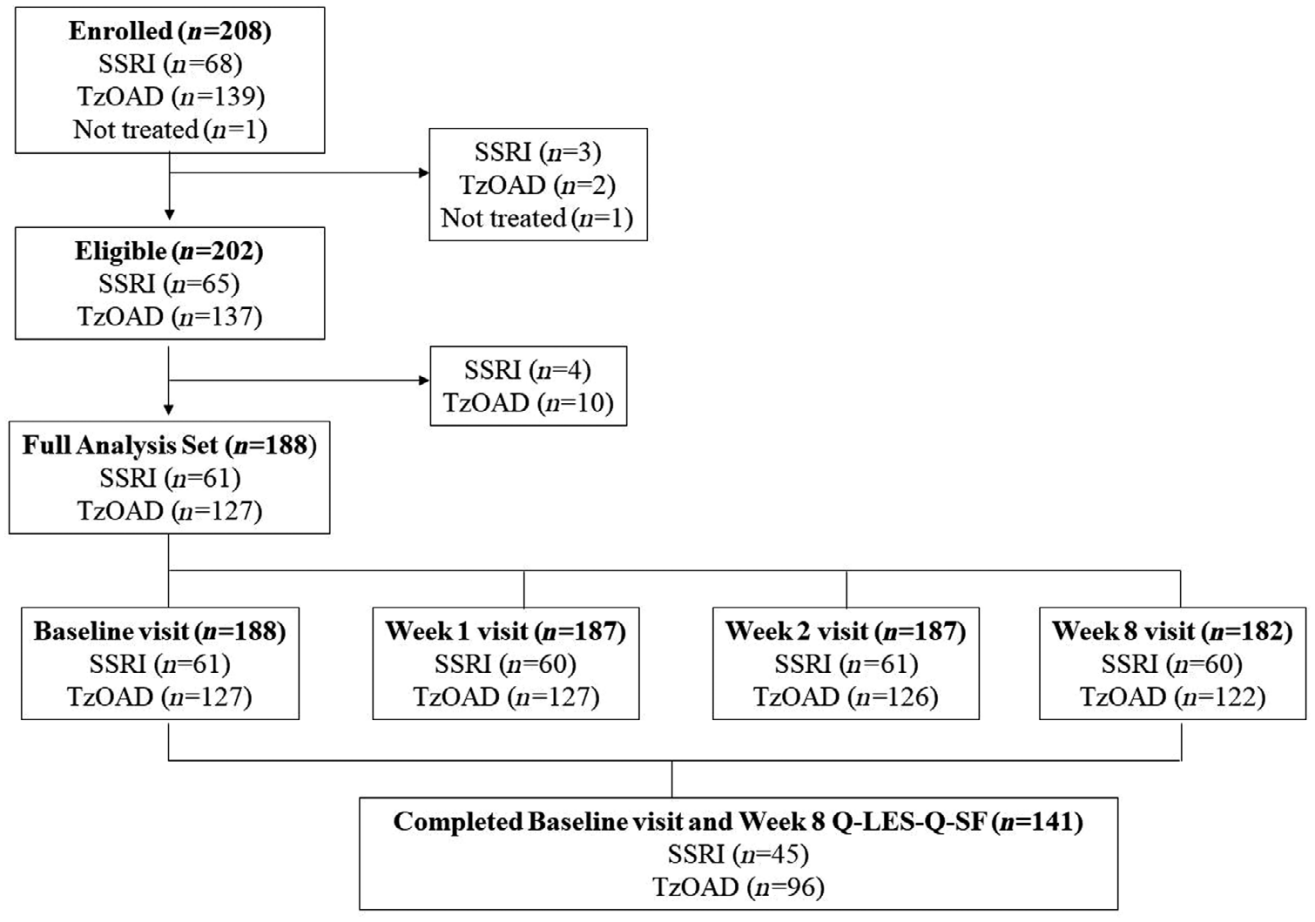
Patient disposition. Q‐LES‐Q‐SF, Quality of Life Enjoyment and Satisfaction Questionnaire Short Form; SSRIs, selective serotonin reuptake inhibitors; TzOAD, trazodone once‐a‐day.

The study included 208 adults with MDD (87 in Bulgaria, 79 in Czech Republic, and 42 in Italy) (Figure 1). Of these, 188 completed the baseline and at least one post‐baseline endpoint assessment. Most of these patients remained on the same treatment as at baseline throughout the study (188 [100%] at week 1, 187 [98.9%] at week 2, and 182 [96.8%] at week 8). Nine patients (4.5%) discontinued between baseline and week 8, of whom seven (5.1%) were in the TzOAD group and two (3.1%) were in the SSRI group (Table S1).

Overall, the mean (standard deviation [SD]) age was 50.2 (14.3) years (Table 2). Most (*n* = 129; 68.6%) patients were female, and most (*n* = 185; 98.4%) were White. About half (*n* = 99; 52.7%) of the patients had experienced previous MDD episodes and, on average among these, each patient had experienced 2.3 previous episodes. Patients had been diagnosed with MDD an average (SD) of 6.7 (10.7) years before enrollment in the study. The mean (SD) duration of the patients’ current depressive episode was 12.1 (24.0) weeks, and depression was moderate on average as measured by MADRS. Nearly two‐thirds of the patients (*n* = 116; 61.7%) had previously received antidepressant treatment, with a mean of 1.3 previous antidepressant treatments. The most common reason for changing antidepressants was a lack of a therapeutic effect (25.5%). Demographics and clinical characteristics were similar between the SSRI and TzOAD groups.

**TABLE 2:** Patient demographic and clinical characteristics.

	SSRI	TzOAD	Overall
	*n* = 61	*n* = 127	*n* = 188
Age (years), mean (SD)	48.2 (15.3)	51.2 (13.7)	50.2 (14.3)
Sex, *n* (%)			
Female	45 (73.8)	84 (66.1)	129 (68.6)
Male	16 (26.2)	43 (33.9)	59 (31.4)
Race, *n* (%)			
White	59 (96.7)	126 (99.2)	185 (98.4)
Black	1 (1.6)	1 (0.8)	2 (1.1)
Other	1 (1.6)	0 (0.0)	1 (0.5)
Time since MDD diagnosis, mean (SD), years	8.2 (11.5)	6.0 (10.4)	6.7 (10.7)
History of previous MDD episodes, *n* (%)	31 (50.8)	68 (53.5)	99 (52.7)
Previously on antidepressant therapy, *n* (%)	37 (60.7)	79 (62.2)	116 (61.7)
Lack of therapeutic effect	8 (15.1)	33 (30.6)	41 (25.5)
Duration of current depressive episode, mean (SD), weeks	7.5 (11.2)	14.4 (27.9)	12.1 (24.0)
Baseline MADRS^a^, mean (SD)	27.8 (5.9)	29.3 (5.5)	28.8 (5.7)

Abbreviations: MADRS, Montgomery Åsberg Depression Rating Scale; MDD, major depressive disorder; SD, standard deviation; SSRI, selective serotonin reuptake inhibitor; TzOAD, trazodone once‐a‐day.

^a^ MADRS total score ranges from 0 to 60; higher scores indicate greater severity of depressive symptoms.

### 3.2 Changes in life enjoyment and satisfaction

**FIGURE 2:**
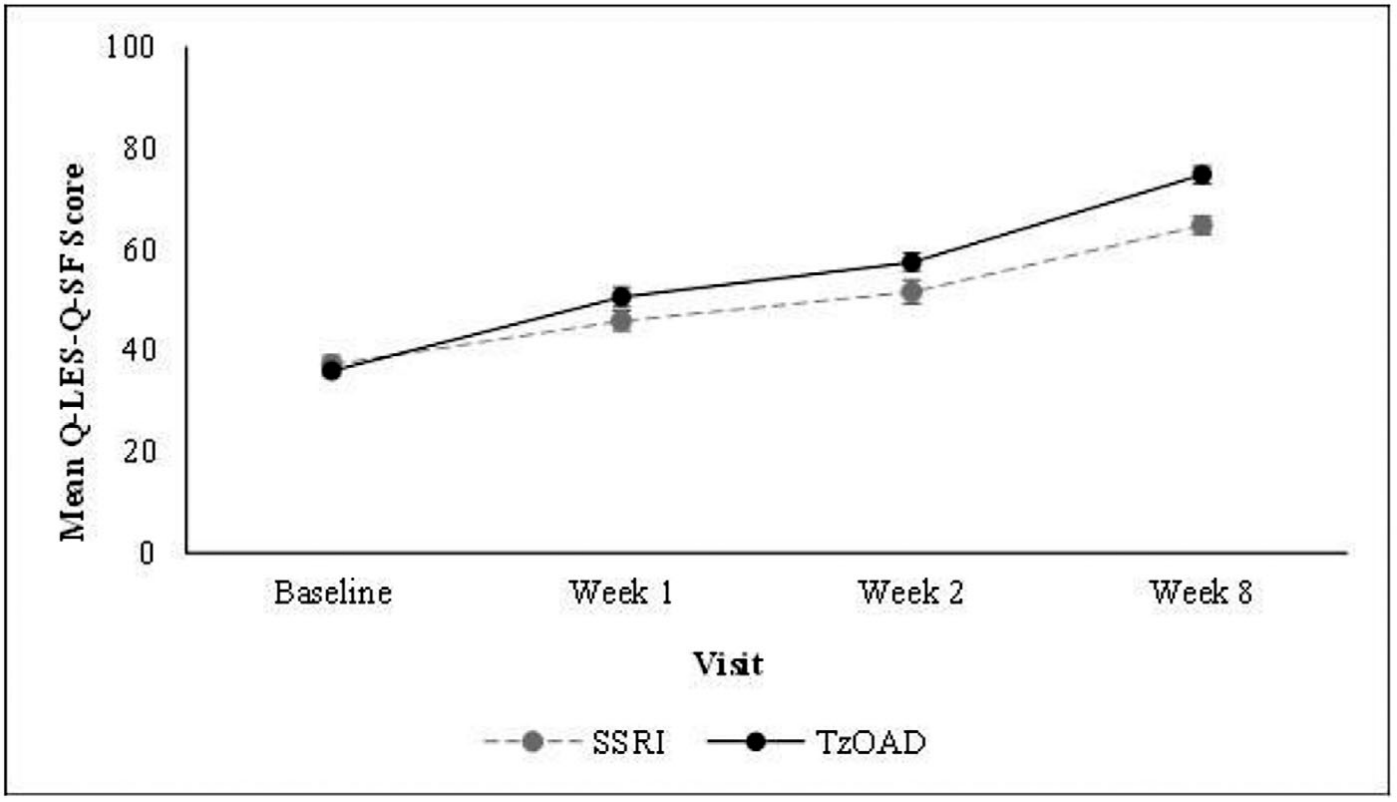
Change in life enjoyment and satisfaction from baseline to week 8 as measured by Q‐LES‐Q‐SF. Values are means ± standard errors. The Q‐LES‐Q score was transformed to a 0–100 scale; scores represent a percentage of the maximum possible score. Higher scores indicate better enjoyment and satisfaction with life. Q‐LES‐Q‐SF, Quality of Life Enjoyment and Satisfaction Questionnaire Short Form; SSRIs, selective serotonin reuptake inhibitors; TzOAD, trazodone once‐a‐day.

Life enjoyment and satisfaction, as measured by the Q‐LES‐Q‐SF, improved steadily over time in both treatment groups, with increases in mean scores detected as early as week 1 (Figure 2; Table S2). At baseline, the mean (SD) Q‐LES‐Q‐SF total score was similar overall (36.4 [12.4]) and in the TzOAD (36.0 [12.4]) and SSRI groups (37.3 [12.5]). At week 8, the mean (SD) Q‐LES‐Q‐SF total score increased to 71.6 (19.7) in the overall group, to 74.8 (18.5) in the TzOAD group, and to 64.8 (20.7) in the SSRI group. In the overall group, the mean change from baseline (SD) was 12.7 (16.7) at week 1, 19.6 (18.3) at week 2, and 35.3 (22.2) at week 8. The mean change in score between baseline and week 8 was 39.0 (22.1) in the TzOAD group and 27.5 (20.4) in the SSRI group.

### 3.3 Changes in cognitive function and individual domains of HRQL

**FIGURE 3:**
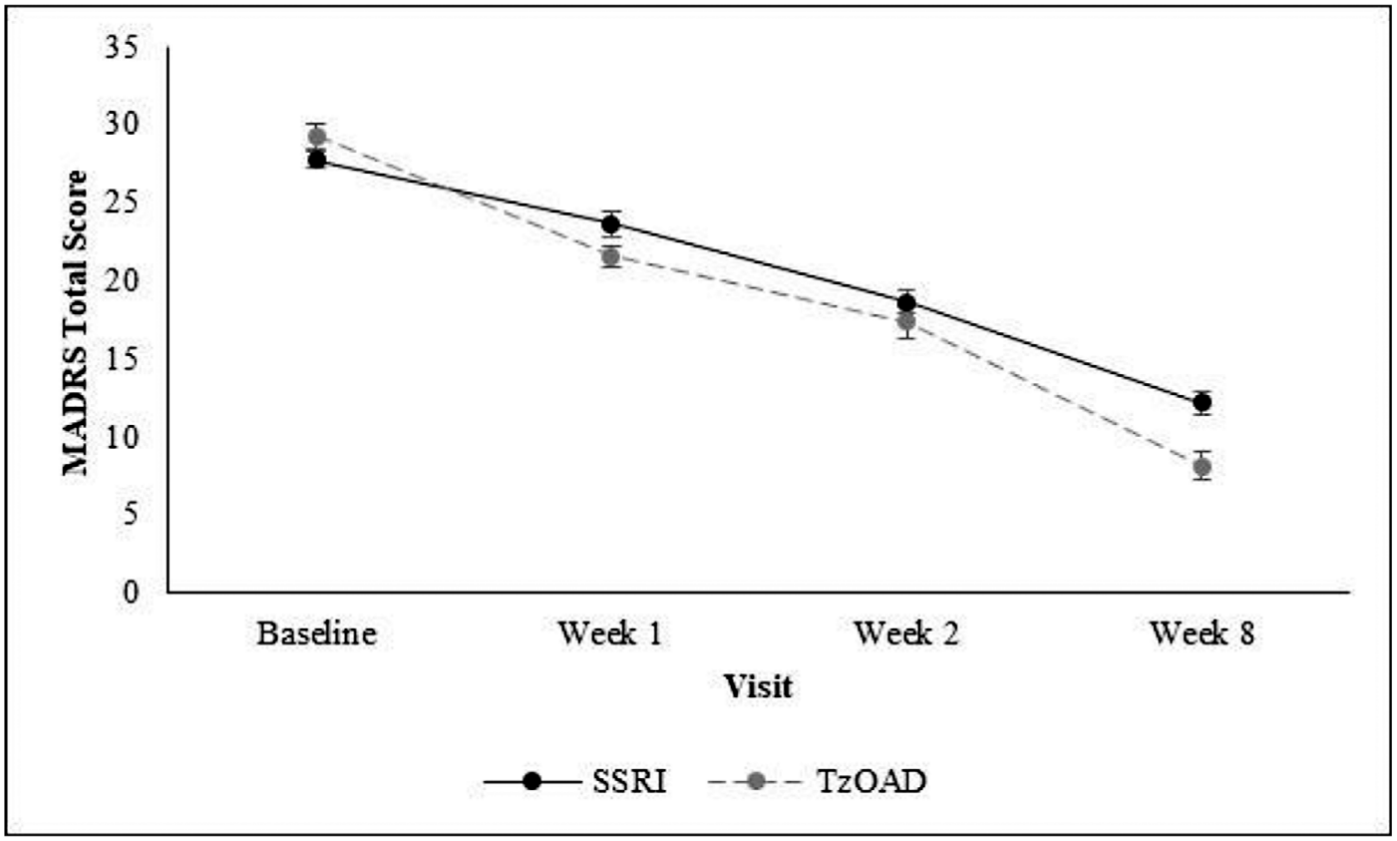
Change in severity of depressive symptoms from baseline to week 8 as measured by MADRS. Values are means ± standard errors. Total scores range from 0 to 60; higher scores indicate greater severity of depressive symptoms. MADRS, Montgomery Åsberg Depression Rating Scale; SSRI, selective serotonin reuptake inhibitor; TzOAD, trazodone once‐a‐day.

**FIGURE 4:**
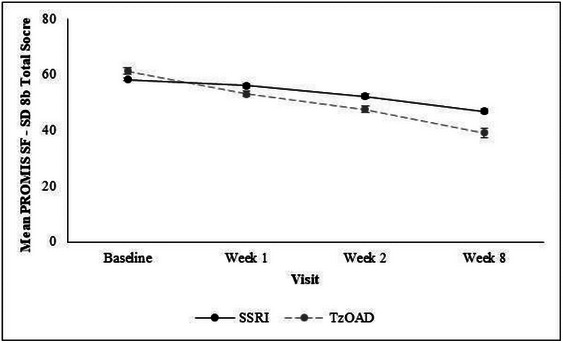
Change in sleep disturbance from baseline to week 8 as measured by PROMIS SF‐SD 8b. Values are means ± standard errors. Total *t*‐score ranges from 28.9 to 76.5; higher scores indicate greater sleep disturbance. PROMIS SF‐SD 8b, Patient‐Reported Outcomes Measurement Information System Sleep Disturbance Short Form 8b; SSRIs, selective serotonin reuptake inhibitors; TzOAD, trazodone once‐a‐day.

Severity of depression, as measured by physicians using the MADRS, improved between baseline and week 8 in both treatment groups (mean [SD] = −15.7 [8.3] in the SSRI group, −21.0 [9.8] in the TzOAD group, and −19.2 [9.7] overall) (Figure 3; Table S3). Sleep disturbance, as measured by PROMIS SF‐SD 8b, also improved from baseline to week 8 in both treatment groups (mean [SD] = −9.9 [12.6] in the SSRI group, −22.0 [12.6] in the TzOAD group, and −18.1 [13.8] overall) (Figure 4; Table S3). For sleep disturbance and severity of depression, improvements in mean scores were detected as early as week 1 in both groups. In addition, between baseline and week 8, patients experienced improved functioning as measured by the SDS (mean [SD] = −9.2 [7.4] in the SSRI group, −14.3 [7.5] in the TzOAD group, and −12.7 [7.9] in the overall group), hedonic capacity as measured by the SHAPS (mean [SD] = −6.6 [4.3] in the SSRI group, −8.3 [4.4] in the TzOAD group, and −7.8 [4.5] in the overall group), and cognitive dysfunction as measured by the PDQ‐5 (mean [SD] = −5.8 [4.5] in the SSRI group, −7.7 [5.0] in the TzOAD group, and −7.1 [4.9] overall) (Table S3).

### 3.4 Association between Q‐LES‐Q‐SF, PROMIS SF‐SD 8b, and MADRS scores

Correlations were significant between the baseline, week 1, and week 2 Q‐LES‐Q‐SF scores and baseline, week 1, and week 2 MADRS and PROMIS SF‐SD 8b scores for the overall sample (Table 3) and for both treatment groups (Tables S4 and S5). Correlations were also significant between weeks 2 and 8 Q‐LES‐Q‐SF scores and weeks 1, 2, and 8 MADRS and PROMIS SF‐SD 8b scores for the overall sample (Table 4) and both treatment groups (Tables S6 and S7). Correlations between score changes in Q‐LES‐Q‐SF, MADRS, and PROMIS SF‐SD 8b scores indicated moderate to strong associations at each post‐baseline visit for the overall sample (Table 5) and for both treatment groups (Tables S8 and S9).

**TABLE 3:** Correlations between Quality of Life Enjoyment and Satisfaction Questionnaire Short Form (Q‐LES‐Q‐SF), Montgomery Åsberg Depression Rating Scale (MADRS), and Patient‐Reported Outcomes Measurement Information System Sleep Disturbance Short Form 8b (PROMIS SF‐SD 8b) scores (overall).

Q‐LES‐Q‐SF1	Baseline				Week 1					Week 2			Week 8			
	MADRS		PROMIS SF‐SD8b^a^		MADRS		PROMIS SF‐SD8b^a^		MADRS		PROMIS SF‐SD8b^a^		MADRS		PROMIS SF‐SD8b^a^	
	N	*r*	N	*r*	N	*r*	N	*r*	N	*r*	N	*r*	N	*r*	N	*r*
Baseline	188	−0.40****	188	−0.29****	186	−0.41****	177	−0.24**	187	−0.35****	173	−0.25**	182	−0.00	141	−0.05
Week 1					177	−0.74****	177	−0.56****	177	−0.57****	167	−0.41****	172	−0.21**	135	−0.38****
Week 2									174	−0.62****	173	−0.59****	170	−0.55****	134	−0.54****
Week 8													141	−0.75****	141	−0.72****

*Note*: For Q‐LES‐Q‐SF, higher numbers indicate a higher level of quality of life, enjoyment, and satisfaction.

^a^ Total score.

Spearman's correlations: *r*—*p < .05; **p < .01; ***p < .001; ****p < .0001.

**TABLE 4:** Correlations between Montgomery Åsberg Depression Rating Scale (MADRS) and Patient‐Reported Outcomes Measurement Information System Sleep Disturbance Short Form 8b (PROMIS SF‐SD 8b) scores.

MADRS	Baseline PROMIS SF‐SD 8b		Week 1 PROMIS SF‐SD 8b		Week 2 PROMIS SF‐SD 8b		Week 8 PROMIS SF‐SD 8b	
	N	*r*	N	*r*	N	*r*	N	R
Baseline	188	0.40****	177	0.34****	173	0.27***	141	0.04
Week 1			177	0.64****	173	0.44****	141	0.29***
Week 2					173	0.62****	141	0.37****
Week 8							141	0.66****

*Note*: For Q‐LES‐Q‐SF, higher numbers indicate a higher level of quality of life, enjoyment, and satisfaction.

Spearman's correlations: *r*—*p < .05; **p < .01; ***p < .001; ****p < .0001. Numbers prior to the correlation coefficients indicate pairwise N‐values.

**TABLE 5:** Correlations between Q‐LES‐Q‐SF, MADRS, and PROMIS SF‐SD 8b score changes

	Week 1				Week 2				Week 8			
	MADRS^1^		PROMIS SF‐SD8b^1^		MADRS^1^		PROMIS SF‐SD8b^1^		MADRS^1^		PROMIS SF‐SD8b^1^	
	N	r	N	r	N	r	N	r	N	r	N	r
**Q‐LES‐Q SF** ^1^												
Week 1	177	‐0.55****	177	‐0.54****								
Week 2					174	‐0.48****	173	‐0.54****				
Week 8									141	‐0.70****	141	‐0.68****
**MADRS** ^1^												
Week 1			177	0.58****								
Week 2							173	0.51****				
Week 8											141	0.64****

Abbreviations: MADRS, Montgomery Åsberg Depression Rating Scale; PROMIS SF‐SD‐8b, Patient‐Reported Outcomes Measurement Information System Sleep Disturbance Short Form 8b; Q‐LES‐Q‐SF, Quality of Life Enjoyment and Satisfaction Questionnaire Short Form

^1^ Total score change from baseline.

Spearman's correlations: r *p<0.05; **p<0.01; ***p<0.001; ****p<0.0001.

### 3.5 Adverse events

Eight patients reported 10 adverse events (AEs) during the study (Table S10). Most AEs were rated as mild (*n* = 8, 80.0%), one AE (10.0%) as moderate, and one AE (10.0%) as severe. Two AEs reported in the TzOAD group and one AE in the SSRI group were considered treatment related. None of the AEs resulted in study discontinuation.

## 4 DISCUSSION

This 8‐week, real‐world evidence study, which included 208 patients with MDD in three European countries, showed that HRQL improved in patients who received TzOAD or SSRI monotherapy. After antidepressant treatment, many aspects of the patients’ lives improved, including sleep disturbance, overall functioning, hedonic capacity, and cognitive function. These improvements could be detected as early as 1 week and continued progressively during the 8 weeks of this study. This paralleled an improvement from moderate to mild depression and a decrease in MADRS scores in both treatment groups (−15.7 in the SSRI group and −21.0 in the TzOAD group) more than the meaningful change threshold of −10 points identified by Hudgens et al. (2021) in patients with treatment‐resistant depression.

The main objective of this study was to evaluate the effect of TzOAD and SSRIs on overall HRQL, as well as individual domains of HRQL, including anhedonia, overall functioning, sleep disturbances, and cognitive function in patients with MDD. All assessed domains of HRQL improved in parallel with overall HRQL. This pattern of findings differs from Sheehan et al. (2011), who reported that patients with MDD or generalized anxiety disorder treated with duloxetine (an SNRI) experienced symptomatic remission more frequently than functional remission, meaning that depressive symptoms and functional symptoms did not always improve in tandem. However, Sheehan et al. (2011) investigated a medication from a different drug class, included a different patient population, and assessed clinician‐reported clinical response instead of patient‐reported mean scores.

This study showed that TzOAD and SSRI were effective in most of the explored variables. During the 8 weeks of this study, life enjoyment and satisfaction as measured by the Q‐LES‐Q‐SF improved more rapidly in the TzOAD group (+108%) than in the SSRI group (+74%). Similar improvements were also found for overall function assessed by the SDS, hedonic capacity assessed by the SHAPS, and cognitive dysfunction assessed by the PDQ‐5. Although the observed numeric differences were not statistically tested, results at different timepoints suggest that improvements in the overall and individual HRQL domains may be larger and occur more quickly with TzOAD.

The current study also examined sleep disturbance as a secondary endpoint. Insomnia afflicts >90% of patients with MDD and is one of the early side effects of SSRIs. Insomnia can also affect the severity and duration of MDD, and poor sleep quality can lead to poor response to treatment (Gebara et al., 2018; Sutton, 2021). Further, insomnia is a risk factor for new onset as well as recurrent episodes of depression (Franzen & Buysse, 2008) Comorbid depression and insomnia increase the duration and severity of depression while increasing the likelihood of depression relapse (Franzen & Buysse, 2008). A 2022 study indicated that the prognosis for MDD is poor in patients with co‐morbid insomnia (Franzen & Buysse, 2008; Xu et al., 2022).

The current study showed that patients had significant sleep disturbance at baseline that progressively improved in both treatment groups. Approximately one‐quarter of patients with MDD discontinue treatment within the first month (Dell'Osso et al., 2020). However, 96% of the patients in the current study completed the full 8 weeks of the study, and >95% adhered to treatment, consistent with the rapid and substantial improvement in HRQL in both treatment groups.

This study provides important real‐world evidence about the effect of TzOAD and SSRIs on HRQL in patients with MDD. Although the treatment groups appeared to be balanced, this study was only observational and descriptive by design and was not powered to detect significant differences  between TzOAD and SSRIs. Moreover, this study did not examine the long‐term changes in HRQL or function. Another potential limitation is that patients were enrolled in only three European countries.

## 5 CONCLUSION

This is the first real‐world study quantitatively evaluating the changes in HRQL in patients with MDD treated with TzOAD. In patients with MDD who received TzOAD or SSRIs, HRQL improved rapidly in parallel with depressive symptoms, which may explain the high treatment adherence in this study. Observed improvements in HRQL and functional parameters that appeared slightly faster and larger in patients treated with TzOAD need to be verified in further research.

## AUTHOR CONTRIBUTIONS

**Valeria Tellone**: Conceptualization; formal analysis; writing—original draft; writing—review and editing. **Oto Markovic**: Investigation; writing—review and editing. **Milena Strashimirova**: Investigation; writing—review and editing. **Gabriele Sani**: Investigation; writing—review and editing. **William R. Lenderking**: Methodology; formal analysis; writing—original draft; writing—review and editing; funding acquisition. **Mary Kay Margolis**: Methodology; funding acquisition; writing—original draft; writing—review and editing; formal analysis. **Raffaella Fallone**: Formal analysis; writing—review and editing. **Elisa Quarchioni**: Formal analysis; writing—review and editing. **Agnese Cattaneo**: Conceptualization; writing—original draft; writing—review and editing. **Alessandro Comandini**: Conceptualization; formal analysis; writing—original draft; writing—review and editing.

## CONFLICT OF INTEREST STATEMENT

Valeria Tellone, Raffaella Fallone, Elisa Quarchioni, Agnese Cattaneo, and Alessandro Comandini are employees of Angelini Pharma S.p.A. Mary Kay Margolis and William R. Lenderking are employees of Evidera Inc. Gabriele Sani received grants or consulting fees from Angelini S.p.A, Lundbeck, Otsuka, Janssen, Neuraxpharm, and Rovi. Oto Markovic has previously worked for Eli Lilly and Company, Bristol‐Myers Squibb, and United Biosource Corporation. Milena Strashimirova declares no conflicts of interest.

### PEER REVIEW

The peer review history for this article is available at https://publons.com/publon/10.1002/brb3.3580.

## Supporting information

Table S1. Percent of patients remaining on treatment at each study visit
Table S2. Change in life enjoyment and satisfaction from baseline to week 8 as measured by Q‐LES‐Q‐SF
Table S3. Change in severity of sleep disturbances and depressive symptoms from baseline to week 8
Table S4. Correlations between Q‐LES‐Q‐SF, MADRS, and PROMIS SF‐SD 8b scores (SSRI)
Table S5. Correlations between Q‐LES‐Q‐SF, MADRS, and PROMIS SF‐SD 8b scores (TzOAD)
Table S6. Correlations between MADRS and PROMIS SF‐SD 8b scores (SSRI)
Table S7. Correlations between MADRS and PROMIS SF‐SD 8b scores (TzOAD)
Table S8. Correlations between Q‐LES‐Q‐SF, MADRS, and PROMIS SF‐SD 8b score changes (SSRI)
Table S9. Correlations between Q‐LES‐Q‐SF, MADRS, and PROMIS SF‐SD 8b score changes (TzOAD)
Table S10. Adverse events
