# Supplementary material for: Impact of trazodone once‐a‐day on quality of life and functional recovery in adults with major depressive disorder: A prospective, observational study
Source: Brain Behav. 2024 Jul 21;14(7):e3580. doi: 10.1002/brb3.3580 (PMC11260556; doi:10.1002/brb3.3580)
Supplement: Supplementary file 1 — Table S1. Percent of patients remaining on treatment at each study visit Table S2. Change in life enjoyment and satisfaction from baseline to week 8 as measured by Q‐LES‐Q‐SF Table S3. Change in severity of sleep disturbances and depressive symptoms from baseline to week 8 Table S4. Correlations between Q‐LES‐Q‐SF, MADRS, and PROMIS SF‐SD 8b scores (SSRI) Table S5. Correlations between Q‐LES‐Q‐SF, MADRS, and PROMIS SF‐SD 8b scores (TzOAD) Table S6. Correlations between MADRS and PROMIS SF‐SD 8b scores (SSRI) Table S7. Correlations between MADRS and PROMIS SF‐SD 8b scores (TzOAD) Table S8. Correlations between Q‐LES‐Q‐SF, MADRS, and PROMIS SF‐SD 8b score changes (SSRI) Table S9. Correlations between Q‐LES‐Q‐SF, MADRS, and PROMIS SF‐SD 8b score changes (TzOAD) Table S10. Adverse events [file BRB3-14-e3580-s001.docx]

# Supplemental Appendix

## Table S1. Percent of patients remaining on treatment at each study visit

|  | **SSRI**  **(N=65)** | **TzOAD**  **(N=137)** | **Overall**  **(N=202)** |
| --- | --- | --- | --- |
| Baseline, n (%) | 65 | 137 | 202 |
| Week 1, n (%) | 64 (98.5) | 135 (98.5) | 199 (98.5) |
| Week 2, n (%) | 64 (98.5) | 134 (97.8) | 198 (98.0) |
| Week 8, n (%) | 63 (96.9) | 130 (94.9) | 193 (95.5) |

Abbreviations: SSRI, selective serotonin reuptake inhibitor; TzOAD, trazodone once-a-day

## Table S2. Change in life enjoyment and satisfaction from baseline to week 8 as measured by Q-LES-Q-SF

|  |  | **SSRI**  **n=61** | |  | **TzOAD**  **n=127** | |  | **Overall**  **n=188** | |
| --- | --- | --- | --- | --- | --- | --- | --- | --- | --- |
|  |  | **N** | **Mean (SD)** |  | **N** | **Mean (SD)** |  | **N** | **Mean (SD)** |
| Q-LES-Q-SF total score^1^ |  |  |  |  |  |  |  |  |  |
| Baseline |  | 61 | 37.3 (12.5) |  | 127 | 36.0 (12.4) |  | 188 | 36.4 (12.4) |
| Week 1 |  | 59 | 45.9 (15.5) |  | 118 | 50.6 (21.2) |  | 177 | 49.0 (19.6) |
| Week 2 |  | 58 | 51.6 (17.9) |  | 116 | 57.5 (19.0) |  | 174 | 55.6 (18.8) |
| Week 8 |  | 45 | 64.8 (20.7) |  | 96 | 74.8 (18.5) |  | 141 | 71.6 (19.7) |
| Q-LES-Q-SF total score change^1^ |  |  |  |  |  |  |  |  |  |
| Baseline to week 1 |  | 59 | 8.5 (11.6) |  | 118 | 14.8 (18.5) |  | 177 | 12.7 (16.7) |
| Baseline to week 2 |  | 58 | 14.2 (15.8) |  | 116 | 22.4 (18.9) |  | 174 | 19.6 (18.3) |
| Baseline to week 8 |  | 45 | 27.5 (20.4) |  | 96 | 39.0 (22.1) |  | 141 | 35.3 (22.2) |

Abbreviations: Q-LES-Q-SF, Quality of Life Enjoyment and Satisfaction Questionnaire Short Form; SD, standard deviation; SSRI, selective serotonin reuptake inhibitor; TzOAD, trazodone once-a-day

^1^The Q-LES-Q score was transformed to a 0–100 scale; scores represent a percentage of the maximum possible score. Higher scores indicate better enjoyment and satisfaction with life.

## Table S3. Change in severity of sleep disturbances and depressive symptoms from baseline to week 8

|  |  |  | **SSRI**  **n=59** |  |  | **TzOAD**  **n=118** |  |  | **Overall**  **N=177** |
| --- | --- | --- | --- | --- | --- | --- | --- | --- | --- |
|  |  | **N** | **Mean (SD)** |  | **N** | **Mean (SD)** |  | **N** | **Mean (SD)** |
| PROMIS SF-SD 8b total score change^1^ |  |  |  |  |  |  |  |  |  |
| Baseline to week 1 |  | 59 | -2.2 (7.3) |  | 118 | -8.1 (9.9) |  | 177 | -6.2 (9.5) |
| Baseline to week 2 |  | 57 | -6.2 (8.8) |  | 116 | -13.6 (10.6) |  | 173 | -11.1 (10.6) |
| Baseline to week 8 |  | 45 | -9.9 (12.6) |  | 96 | -22.0 (12.6) |  | 141 | -18.1 (13.8) |
|  |  |  |  |  |  |  |  |  |  |
| MADRS total score change^2^ |  |  |  |  |  |  |  |  |  |
| Baseline to week 1 |  | 60 | -4.0 (5.4) |  | 126 | -7.7 (8.1) |  | 186 | -6.5 (7.6) |
| Baseline to week 2 |  | 61 | -9.0 (8.1) |  | 126 | -11.9 (7.8) |  | 187 | -10.9 (8.0) |
| Baseline to week 8 |  | 60 | -15.7 (8.3) |  | 122 | -21.0 (9.8) |  | 182 | -19.2 (9.7) |
|  |  |  |  |  |  |  |  |  |  |
| SDS total score change^3^ |  | 43 | -9.2 (7.4) |  | 92 | -14.3 (7.5) |  | 135 | -12.7 (7.9) |
| SHAPS total score change^4^ |  | 44 | -6.6 (4.3) |  | 94 | -8.3 (4.4) |  | 138 | -7.8 (4.5) |
| PDQ-5 total score change^5^ |  | 44 | -5.8 (4.5) |  | 94 | -7.7 (5.0) |  | 138 | -7.1 (4.9) |

Abbreviation: MADRS, Montgomery Åsberg Depression Rating Scale; PDQ, Perceived Deficits Questionnaire; PROMIS SF-SD 8b, Patient-Reported Outcomes Measurement Information System Sleep Disturbance Short Form 8b; SD, standard deviation; SDS, Sheehan Disability Scale; SHAPS, Snaith-Hamilton Pleasure Scale; SSRI, selective serotonin reuptake inhibitor; TzOAD, trazodone once-a-day

^1^Total t-score ranges from 28.9–76.5; higher scores indicate greater sleep disturbance.

^2^Total score ranges from 0–60; higher scores indicate greater severity of depressive symptoms.

^3^Total score ranges from 0–30; higher scores indicate greater functional impairment.

^4^Total score ranges from 0–14; higher scores indicate greater present state of anhedonia.

^5^Total score ranges from 0–20; higher scores indicate perceived cognitive dysfunction.

## Table S4. Correlations between Q-LES-Q-SF, MADRS, and PROMIS SF-SD 8b scores (SSRI)

|  | **Baseline** | | | | **Week 1** | | | |  | **Week 2** | | | **Week 8** | | | |
| --- | --- | --- | --- | --- | --- | --- | --- | --- | --- | --- | --- | --- | --- | --- | --- | --- |
| **Q-LES-Q SF1** | **MADRS** | | **PROMIS SF-SD8b^1^** | | **MADRS** | | **PROMIS SF-SD8b^1^** | | **MADRS** | | **PROMIS SF-SD8b^1^** | | **MADRS** | | **PROMIS SF-SD8b^1^** | |
|  | **N** | **r** | **N** | **r** | **N** | **r** | **N** | **r** | **N** | **r** | **N** | **r** | **N** | **r** | **N** | **r** |
| Baseline | 61 | -0.39** | 61 | -0.36** | 60 | -0.38** | 59 | -0.30 | 61 | -0.24 | 57 | -0.28* | 60 | -0.28* | 45 | -0.32* |
|  |  |  |  |  |  |  |  |  |  |  |  |  |  |  |  |  |
| Week 1 |  |  |  |  | 59 | - 0.63**** | 59 | - 0.55**** | 59 | ‑0.53**** | 56 | -0.44*** | 58 | -0.33* | 44 | -0.36* |
|  |  |  |  |  |  |  |  |  |  |  |  |  |  |  |  |  |
| Week 2 |  |  |  |  |  |  |  |  | 58 | -0.64**** | 57 | - 0.58**** | 57 | - 0.52**** | 43 | -0.49** |
|  |  |  |  |  |  |  |  |  |  |  |  |  |  |  |  |  |
| Week 8 |  |  |  |  |  |  |  |  |  |  |  |  | 45 | -0.76**** | 45 | - 0.63**** |

Abbreviations: MADRS, Montgomery Åsberg Depression Rating Scale; PROMIS SF-SD-8b, Patient-Reported Outcomes Measurement Information System Sleep Disturbance Short Form 8b; Q-LES-Q-SF, Quality of Life Enjoyment and Satisfaction Questionnaire Short Form; SSRI, selective serotonin reuptake inhibitor

For Q-LES-Q-SF, higher numbers indicate a higher level of quality of life, enjoyment, and satisfaction.

^1^Total score.

Spearman’s correlations: r *p<0.05; **p<0.01; ***p<0.001; ****p<0.0001.

## Table S5. Correlations between Q-LES-Q-SF, MADRS, and PROMIS SF-SD 8b scores (TzOAD)

|  | **Baseline** | | | | **Week 1** | | | |  | **Week 2** | | | **Week 8** | | | |
| --- | --- | --- | --- | --- | --- | --- | --- | --- | --- | --- | --- | --- | --- | --- | --- | --- |
| **Q-LES-Q SF1** | **MADRS** | | **PROMIS SF-SD8b^1^** | | **MADRS** | | **PROMIS SF-SD8b^1^** | | **MADRS** | | **PROMIS SF-SD8b^1^** | | **MADRS** | | **PROMIS SF-SD8b^1^** | |
|  | **N** | **r** | **N** | **r** | **N** | **r** | **N** | **r** | **N** | **r** | **N** | **r** | **N** | **r** | **N** | **r** |
| Baseline | 127 | -0.39**** | 127 | -0.25** | 126 | -0.43**** | 118 | -0.23* | 126 | -0.41**** | 116 | -0.26** | 122 | 0.12 | 96 | 0.05 |
|  |  |  |  |  |  |  |  |  |  |  |  |  |  |  |  |  |
| Week 1 |  |  |  |  | 118 | -0.79**** | 118 | -0.57**** | 118 | -0.60**** | 111 | -0.41**** | 114 | -0.12 | 91 | -0.38*** |
|  |  |  |  |  |  |  |  |  |  |  |  |  |  |  |  |  |
| Week 2 |  |  |  |  |  |  |  |  |  |  |  |  |  |  |  |  |
|  |  |  |  |  |  |  |  |  | 116 | -0.60**** | 116 | -0.57**** | 113 | -0.55**** | 91 | -0.52**** |
|  |  |  |  |  |  |  |  |  |  |  |  |  |  |  |  |  |
| Week 8 |  |  |  |  |  |  |  |  |  |  |  |  | 96 | -0.73**** | 96 | -0.72**** |

Abbreviations: MADRS, Montgomery Åsberg Depression Rating Scale; PROMIS SF-SD-8b, PROMIS Short Form - Sleep Disturbance - 8b; Q-LES-Q-SF, Quality of Life Enjoyment and Satisfaction Questionnaire Short Form; TzOAD, trazodone once-a-day

For Q-LES-Q-SF, higher numbers indicate a higher level of quality of life, enjoyment, and satisfaction.

^1^Total score.

Spearman’s correlations: r *p<0.05; **p<0.01; ***p<0.001; ****p<0.0001.

## Table S6. Correlations between MADRS and PROMIS SF-SD 8b scores (SSRI)

| **MADRS** | **Baseline**  **PROMIS SF-SD 8b** | | **Week 1**  **PROMIS SF-SD 8b** | | **Week 2**  **PROMIS SF-SD 8b** | | **Week 8**  **PROMIS SF-SD 8b** | |
| --- | --- | --- | --- | --- | --- | --- | --- | --- |
|  | **N** | **r** | **N** | **r** | **N** | **r** | **N** | **r** |
| Baseline | 61 | 0.43*** | 59 | 0.39** | 57 | 0.28* | 45 | 0.19 |
| Week 1 |  |  | 59 | 0.68**** | 57 | 0.59**** | 45 | 0.44** |
| Week 2 |  |  |  |  | 57 | 0.73**** | 45 | 0.51*** |
| Week 8 |  |  |  |  |  |  | 45 | 0.73**** |

Abbreviation: MADRS, Montgomery Åsberg Depression Rating Scale; PROMIS SF-SD 8b, Patient-Reported Outcomes Measurement Information System Sleep Disturbance Short Form 8b; SSRI, selective serotonin reuptake inhibitor

Spearman's correlation: r *p<0.05; **p<0.01; ***p<0.001; ****p<0.0001. Numbers prior to the correlation coefficients indicate pairwise ns.

## Table S7. Correlations between MADRS and PROMIS SF-SD 8b scores (TzOAD)

| **MADRS** | **Baseline**  **PROMIS SF-SD 8b** | | **Week 1**  **PROMIS SF-SD 8b** | | **Week 2**  **PROMIS SF-SD 8b** | | **Week 8**  **PROMIS SF-SD 8b** | |
| --- | --- | --- | --- | --- | --- | --- | --- | --- |
|  | **N** | **r** | **N** | **r** | **N** | **r** | **N** | **r** |
| Baseline | 127 | 0.37**** | 118 | 0.35**** | 116 | 0.33*** | 96 | 0.01 |
| Week 1 |  |  | 118 | 0.63**** | 116 | 0.40**** | 96 | 0.22* |
| Week 2 |  |  |  |  | 116 | 0.60**** | 96 | 0.31** |
| Week 8 |  |  |  |  |  |  | 96 | 0.57**** |

Abbreviation: MADRS, Montgomery Åsberg Depression Rating Scale; PROMIS SF-SD 8b, Patient-Reported Outcomes Measurement Information System Sleep Disturbance Short Form 8b; TzOAD, trazodone once-a-day

Spearman's correlations: r *p<0.05; **p<0.01; ***p<0.001; ****p<0.0001. Numbers prior to the correlation coefficients indicate pairwise ns.

## Table S8. Correlations between Q-LES-Q-SF, MADRS, and PROMIS SF-SD 8b score changes (SSRI)

|  | **Week 1** | | | | **Week 2** | | | | **Week 8** | | | |
| --- | --- | --- | --- | --- | --- | --- | --- | --- | --- | --- | --- | --- |
|  | **MADRS^1^** | | **PROMIS SF-SD8b^1^** | | **MADRS^1^** | | **PROMIS SF-SD8b^1^** | | **MADRS^1^** | | **PROMIS SF-SD8b^1^** | |
|  | **N** | **r** | **N** | **r** | **N** | **r** | **N** | **r** | **N** | **r** | **N** | **r** |
| **Q-LES-Q SF^1^** | | |  |  |  |  |  |  |  |  |  |  |
| Week 1 | 59 | -0.45*** | 59 | -0.48*** |  |  |  |  |  |  |  |  |
| Week 2 |  |  |  |  | 58 | -0.45*** | 57 | -0.54**** |  |  |  |  |
| Week 8 |  |  |  |  |  |  |  |  | 45 | -0.68**** | 45 | -0.61**** |
|  |  |  |  |  |  |  |  |  |  |  |  |  |
| **MADRS^1^** |  |  |  |  |  |  |  |  |  |  |  |  |
| Week 1 |  |  | 59 | 0.51**** |  |  | 57 | 0.48*** |  |  |  |  |
| Week 2 |  |  |  |  |  |  |  |  |  |  |  |  |
| Week 8 |  |  |  |  |  |  |  |  |  |  | 45 | 0.69**** |

Abbreviations: MADRS, Montgomery Åsberg Depression Rating Scale; PROMIS SF-SD-8b, Patient-Reported Outcomes Measurement Information System Sleep Disturbance Short Form 8b; Q-LES-Q-SF, Quality of Life Enjoyment and Satisfaction Questionnaire Short Form; SSRI, selective serotonin reuptake inhibitor

^1^Total score change from baseline.

Spearman's correlations: r *p<0.05; **p<0.01; ***p<0.001; ****p<0.0001.

## Table S9. Correlations between Q-LES-Q-SF, MADRS, and PROMIS SF-SD 8b score changes (TzOAD)

|  | **Week 1** | | | | **Week 2** | | | | **Week 8** | | | |
| --- | --- | --- | --- | --- | --- | --- | --- | --- | --- | --- | --- | --- |
|  | **MADRS^1^** | | **PROMIS SF-SD8b^1^** | | **MADRS^1^** | | **PROMIS SF-SD8b^1^** | | **MADRS^1^** | | **PROMIS SF-SD8b^1^** | |
|  | **N** | **r** | **N** | **r** | **N** | **r** | **N** | **r** | **N** | **r** | **N** | **r** |
| **Q-LES-Q SF^1^** | | |  |  |  |  |  |  |  |  |  |  |
| Week 1 | 118 | -0.57**** | 118 | -0.56**** |  |  |  |  |  |  |  |  |
| Week 2 |  |  |  |  | 116 | -0.44**** | 116 | -0.47**** |  |  |  |  |
| Week 8 |  |  |  |  |  |  |  |  | 96 | -0.65**** | 96 | -0.64**** |
|  |  |  |  |  |  |  |  |  |  |  |  |  |
| **MADRS^1^** |  |  |  |  |  |  |  |  |  |  |  |  |
| Week 1 |  |  | 118 | 0.61**** |  |  | 116 | 0.47**** |  |  |  |  |
| Week 2 |  |  |  |  |  |  |  |  |  |  |  |  |
| Week 8 |  |  |  |  |  |  |  |  |  |  | 96 | 0.53**** |

Abbreviation: MADRS, Montgomery Åsberg Depression Rating Scale; PROMIS SF-SD 8b, Patient-Reported Outcomes Measurement Information System Sleep Disturbance Short Form 8b; Q-LES-Q-SF, Quality of Life Enjoyment and Satisfaction Questionnaire Short Form; TzOAD, trazodone once-a-day

^1^Total score change from baseline.

Spearman's correlations: r *p<0.05; **p<0.01; ***p<0.001; ****p<0.0001.

## Table S10. Adverse events

|  | **SSRI**  **N=65** | **TzOAD**  **N=137** | **Overall**  **N=202** |
| --- | --- | --- | --- |
| Patients with at least one AE, n (%) | 3 (4.6) | 5 (3.6) | 8 (4.0) |
| Total number of AEs reported during study | 4 | 6 | 10 |
| Type of treatment-emergent AEs (n, %) |  |  |  |
| Gastrointestinal disorders |  |  |  |
| Constipation | 0 (0) | 1 (16.7) | 1 (10.0) |
| Nausea | 1 (25.0) | 0 (0) | 1 (10.0) |
| Infections and infestations |  |  |  |
| COVID-19 | 2 (50.0) | 0 (0) | 2 (20.0) |
| Injury, poisoning, and procedural complications |  |  |  |
| Exposure to SARS-CoV-2* | 0 (0) | 1 (16.7) | 1 (10.0) |
| Investigations |  |  |  |
| Body temperature increased | 0 (0) | 1 (16.7) | 1 (10.0) |
| Nervous system disorders |  |  |  |
| Balance disorder | 0 (0) | 1 (16.7) | 1 (10.0) |
| Psychiatric disorders |  |  |  |
| Hallucination | 0 (0) | 1 (16.7) | 1 (10.0) |
| Skin and subcutaneous tissue disorders |  |  |  |
| Dermal cyst | 1 (25.0) | 0 (0) | 1 (10.0) |
| Social circumstances |  |  |  |
| Death of relative** | 0 (0) | 1 (16.7) | 1 (10.0) |
| AE intensity, n (%) |  |  |  |
| Mild | 4 (100.0) | 4 (66.7) | 8 (80.0) |
| Moderate | 0 (0) | 1 (16.7) | 1 (10.0) |
| Severe | 0 (0) | 1 (16.7) | 1 (10.0) |
| Treatment-related, n (%) |  |  |  |
| Yes | 1 (25.0) | 2 (33.3) | 3 (30.0) |
| No | 3 (75.0) | 4 (66.7) | 7 (70.0) |

Abbreviations: AE, adverse events; COVID-19, coronavirus disease of 2019; SARS-CoV-2, severe acute respiratory syndrome coronavirus 2; SSRI, selective serotonin reuptake inhibitor; TzOAD, trazodone once-a-day

*Moderate AE. **Severe AE.
